# Characterization of *Clostridioides difficile* Strains from an Outbreak Using MALDI-TOF Mass Spectrometry

**DOI:** 10.3390/microorganisms10071477

**Published:** 2022-07-21

**Authors:** Adriana Calderaro, Mirko Buttrini, Benedetta Farina, Sara Montecchini, Monica Martinelli, Maria Cristina Arcangeletti, Carlo Chezzi, Flora De Conto

**Affiliations:** 1 Department of Medicine and Surgery, University of Parma, Viale A. Gramsci 14, 43126 Parma, Italy; mirko.buttrini@unipr.it (M.B.); benedetta.farina@unipr.it (B.F.); mariacristina.arcangeletti@unipr.it (M.C.A.); carlo.chezzi@unipr.it (C.C.); flora.deconto@unipr.it (F.D.C.); 2 Unit of Clinical Virology, University Hospital of Parma, Viale A. Gramsci 14, 43126 Parma, Italy; smontecchini@ao.pr.it; 3 Unit of Clinical Microbiology, University Hospital of Parma, Viale A. Gramsci 14, 43126 Parma, Italy; mmartinelli@ao.pr.it

**Keywords:** outbreak, *Clostridioides difficile*, MALDI-TOF MS

## Abstract

The epidemiology of *Clostridioides difficile* infection (CDI) has changed over the last two decades, due to the emergence of *C. difficile* strains with clinical relevance and responsible for nosocomial outbreaks with severe outcomes. This study reports an outbreak occurred in a Long-term Care Unit from February to March 2022 and tracked by using a Matrix-Assisted Desorption/Ionization Time-of-Flight Mass Spectrometry (MALDI-TOF MS) typing approach (T-MALDI); subsequently, a characterization of the toxigenic and antimicrobial susceptibility profiles of the *C. difficile* isolates was performed. A total of 143 faecal samples belonging to 112 patients was evaluated and *C. difficile* DNA was detected in 51 samples (46 patients). Twenty-nine *C. difficile* isolates were obtained, and three different clusters were revealed by T-MALDI. The most representative cluster accounted 22 strains and was considered to be epidemic, in agreement with PCR-Ribotyping. Such epidemic strains were susceptible to vancomycin (MIC ≤ 0.5 mg/mL) and metronidazole (MIC ≤ 1 mg/mL), but not to moxifloxacin (MIC > 32 mg/mL). Moreover, they produced only the Toxin A and, additionally, the binary toxin. To our knowledge, this is the first reported outbreak referable to a *tcd*A+/*tcd*B-/*cdt*+ genotypic profile. In light of these results, T-MALDI is a valid and rapid approach for discovering and tracking outbreaks.

## 1. Introduction

*Clostridioides difficile* is the leading cause of nosocomial diarrhoea in industrialized countries and the aetiological agent of antibiotic-associated pseudomembranous colitis [1,2,3]. The clinical spectrum of *C. difficile* infection (CDI) ranges from mild, self-limiting diarrhoea to severe outcomes such as fulminant colitis, toxic megacolon, bowel perforation, and sepsis [3,4].

With regard to the pathogenesis, the CDI occurs through a toxin-mediated mechanism involving the enterotoxin A (TcdA) and the cytotoxin B (TcdB), both able to induce intestinal mucosal damage and local inflammation [5,6].

The severity of such disease could be enhanced through the expression of the binary toxin (CDT), an additional virulence factor frequently observed in *C. difficile* epidemic strains, especially those responsible for hospital outbreaks [7,8]. Moreover, the most severe CDI cases with poor outcomes could also be associated with loss/alteration of the negative regulator factor (TcdC) of the *tcd*A and *tcd*B toxin-encoding genes, as well as with high-level fluoroquinolone resistance [7,9]. Moreover, these strains show with greater frequency, a reduced susceptibility to the first-line antibiotics (vancomycin and metronidazole) used for CDI treatment and/or for relapsing infections [10,11].

The spread of *C. difficile* strains carrying such hypervirulent properties has led to increased rates of morbidity and mortality over the last two decades [5,9,12].

In particular, during the early 2000s, the epidemiology of CDI deeply changed worldwide since the emergence of the BI/NAP1/027 strain, whose rapid spread resulted in the burden of nosocomial outbreaks associated with high mortality rates and a greater risk of relapse [9].

Currently, BI/NAP1/027 continues to be the predominant *C. difficile* nosocomial epidemic strain in U.S.A. [13], whereas the majority of clinical toxigenic strains isolated in European hospital settings belong to the 078/126 type [14].

Despite the wide diffusion of these two epidemic strains worldwide, recent European epidemiological data report the emergence and the increasing spread of new virulent genotypes, able to cause outbreaks in hospitals and health-care settings with associated severe outcomes [9,12,14,15].

For instance, the 018 and 607 types have recently been reported as the leading cause of nosocomial CDIs in Italy, according to an epidemiological ten-year (2006–2016)-period study performed by the reference Central Laboratory Service for *C. difficile* of Istituto Superiore di Sanità (ISS-CLSCD) [12]. In particular, these two Italian epidemic strains were significantly associated to the CDI outbreaks occurred in Critical Care Departments, General Medicine Units, Clinic Units, and Long-term Care Facilities with reported higher rate of severe outcomes [12,15].

Given the high diffusion rate and interpatient transmissibility observed for virulent *C. difficile* types such as 018 and 607, a CDI microbiological surveillance network is urgently needed in order to better monitor the distribution of CDI at a regional, national, and international level, thus avoiding to underestimate the real epidemiological scenario and the related incidence, severity, and mortality rates [12,15]. The epidemiological tracking of CDI relies on molecular typing methods, such as restriction endonuclease analysis (REA), pulsed-field gel electrophoresis (PFGE), capillary or conventional agarose gel-based polymerase chain reaction (PCR) Ribotyping (PCR-Ribotyping), MultiLocus Variable-number tandem repeat analysis (MLVA), and MultiLocus sequence typing (MLST), as well as whole-genome sequencing (WGS) and, more recently, Matrix-Assisted Desorption/Ionization-Time of Flight Mass Spectrometry (MALDI-TOF MS) [16,17]. PCR-Ribotyping and PFGE are the most used genotyping methods adopted in Europe and Northern America, respectively; in particular, PCR-Ribotyping characterizes different *C. difficile* strains by the amplification of the Intergenic Spacer Region (ISR), located between 16S and 23S ribosomal genes, which has intraspecific high variability in terms of both length and nucleotide sequence; therefore, its variations identify different ribotypes [16,17,18]. MALDI-TOF MS for *C. difficile* classification was proposed as an alternative to PCR-Ribotyping. In particular, the protein spectra acquisition for MALDI-TOF MS typing has been proved to be easier, faster, and cheaper compared to PCR-Ribotyping and also suitable for a single-strain analysis [16].

The aim of this study was the typing of *C. difficile* isolates from a suspected outbreak lasted 2 months and occurred in a Long-term Care Unit and in an Internal Medicine ward by using a MALDI-TOF MS approach, compared to the PCR-Ribotyping. Moreover, a characterization of the toxigenic and susceptibility profiles of the *C. difficile* isolates was also performed.

## 2. Materials and Methods

### 2.1. Diagnostic Algorithm

A total of 143 faecal samples belonging to 112 patients (72 females and 40 males; mean age 81 years ranging from 36 to 101 years) attending a Long-term care Unit and an Internal Medicine ward, with suspicion of CDI, as reported in the medical order, were included in this study (Table 1). All the samples were sent to the Unit of Clinical Microbiology of the University Hospital of Parma (Italy) during a 4-month period (from December 2021 to March 2022) for diagnostic purposes. Laboratory diagnosis was performed upon medical order and a clinical report was produced. Anonymization of the samples was done before data analysis and medical information was protected.

All stool samples were prospectively analysed for the detection of toxigenic *C. difficile* by a two-step diagnostic algorithm, as previously described [16]. Briefly, the first step involved a molecular qualitative assay (Illumigene^TM^ *C. difficile*, Meridian Bioscience, Cincinnati, OH, USA), based on a loop-mediated isothermal DNA amplification (LAMP) technology, able to detect toxigenic *C. difficile* by amplifying a conserved 204 bp nucleotide sequence inside *C. difficile*’s PaLoc, that is located at the 5′ region of the *tcd*A gene. The assay was performed according to the manufacturer’s instructions, as previously described [16,19]. The second step, performed only on toxigenic *C. difficile* DNA positive samples, involved the simultaneous detection of the glutamate dehydrogenase enzyme (GDH) and of the toxins A/B by an immunochromatographic assay (C. DIFF QUICK CHECK COMPLETE TechLab, Blacksburg, VA, USA), performed according to the manufacturer’s instructions as previously described [16,19]. In parallel, the samples were submitted to *C. difficile* isolation by conventional culture (CC). Briefly, an aliquot of faecal sample was added to an enrichment medium (Cooked meat broth, Kima, Padova, Italy), incubated at 37 °C in anaerobic conditions (95% N_2_, 5% CO_2_) for 72 h, and then heat-shocked (100 °C for 3 min) before plating onto a specific selective medium (cycloserine-cefoxitin-fructose agar—CCFA, Kima). After incubation at 37 °C in anaerobic conditions for at least 48 h, the species identification of putative *C. difficile* colonies was performed by a MALDI-TOF mass spectrometer (Bruker, Bremen, Germany) [16,19].

A 2 McFarland suspension in 1 mL of sterile double-distilled water of each *C. difficile* isolate from CCFA culture was used for the detection of the genescodifying the A, B, and CDT toxins (*tcd*A, *tcd*B, and *cdt*A/*cdt*B, respectively) and to typing both by MALDI-TOF MS and PCR-Ribotyping.

For the detection of *tcd*A, *tcd*B, and *cdt*A/*cdt*B genes and for PCR-Ribotyping, an aliquot of 400 µL of the bacterial suspension was treated by heat-shock at 100 °C for 10 min [16,19] and the supernatant containing *C. difficile* DNA was stored at 4 °C until the amplification.

For MALDI-TOF MS typing, an aliquot of 300 µL was submitted to protein extraction as previously described [16].

### 2.2. Detection of C. difficile tcdA, tcdB, and cdtA/cdtB Genes

Two different sets of previously described [20] specific primers (Biosense, Milan, Italy) TA1 (5′-ATG ATA AGG CAA CTT CAG TGG-3′)/TA2 (5′-TAA GTT CCT CCT GCT CCA TCA A-3′) and TB1 (5′-GAG CTG CTT CAA TTG GAG AGA-3′)/TB2 (5′-GTA ACC TAC TTT CAT AAC ACC AG-3′) were used to amplify the *tcd*A and *tcd*B genes, respectively. The amplification reaction was performed according to Spigaglia et al. [20] with some modifications: an aliquot of 10 µL of *C. difficile* DNA was added to a 40-µL reaction mixture containing PCR Buffer 1X(5 µL) (Roche, Monza, Italy), 1.5 mM MgCl_2_ (Roche), 10 pmol of TA1 and TA2 primers, 5 pmol of TB1 and TB2 primers, 200 µM of dNTPs (Roche), and 1.5 U TaqDNA polymerase (Roche). The amplification was carried out in a GeneAmp PCR System 9700 thermalcycler (Applied Biosystems, Foster City, CA, USA), according to the following protocol: one cycle of 2 min at 95 °C; 30 cycles of 30 s at 95 °C, 30 s at 60 °C, and 30 s at 72 °C, and a final extension cycle of 5 min at 72 °C.

Two different sets of previously described [21] specific primers (Biosense) cdtApos (5′-TGA ACC TGG AAA AGG TGA TG-3′)/cdtArev (5′-AGG ATT ATT TAC TGG ACC ATT TG-3′) and cdtBpos (5′-CTT AAT GCA AGT AAA TAC TGA G-3′)/cdtBrev (5′-AAC GGA TCT CTT GCT TCA GTC-3′) were used to amplify *cdt*A and *cdt*B genes, respectively. Amplification reaction was performed according to Stubbs et al. [21] with some modifications: an aliquot of 10 µL of *C. difficile* DNA was added to a 40-µL reaction mixture containing PCR Buffer 1X (5 µL) (Roche), 1.5 mM MgCl_2_ (Roche), 0.3 µM of cdtApos and cdtArev primers, or 0.4 µM of cdtBpos and cdtBrev primers, 200 µM of dNTPs (Roche), and 1.5 U TaqDNA polymerase (Roche). The amplification was carried out in a GeneAmp PCR System 9700 thermalcycler (Applied Biosystems), according to the following protocol: one cycle of 2 min at 95 °C; 30 cycles of 1 min at 94 °C, 1 min at 52 °C, and 1 min and 20 s at 72 °C; and a final extension cycle of 5 min at 72 °C.

Amplification products (10 µL added to 2 µL of bromophenol blue, Invitrogen, Paisley, UK) were separated by electrophoresis through a 1% agarose gel in Tris-acetate-EDTA buffer for 1 h at 100 V and revealed on a UV table after GelRed^®^ staining. Gel images were acquired digitally.

### 2.3. PCR-Ribotyping

The ISR was amplified by using two specific primers (Eurogentec, Seraing, Belgium), previously described [22]: RtFR1 (5′-GTG CGG CTG GAT CAC CTC CT-3′) complementary to the 3′ terminal region of the 16S ribosomal gene and RtFR2 (5′-CCC TGC ACC CTT AAT AAC TTG ACC-3′) complementary to the 5′ terminal region of the 23S ribosomal gene. Amplification reaction was performed according to Bidet et al. [22], with some modifications [16]. Briefly, an aliquot of 15 µL of *C. difficile* DNA was added to a 35-µL reaction mixture containing PCR Buffer 1X (5 µL) (Roche, Monza, Italy), 1.5 mM MgCl_2_ (Roche), 10 pmol of each primer, 200 µM of dNTPs (Roche), and 1.5 U TaqDNA polymerase (Roche). The amplification was carried out in a GeneAmp PCR System 9700 thermalcycler (Applied Biosystems, Foster City, CA, USA), according to the following protocol: one cycle of 6 min at 95 °C; 35 cycles of 1 min at 94 °C, 1 min at 57 °C, and 2 min at 72 °C; and a final extension cycle of 7 min at 72 °C. Amplification products (10 µL added to 2 µL of bromophenol blue, Invitrogen, Paisley, UK) were separated by electrophoresis through a 3% agarose gel in Tris-acetate-EDTA buffer for 5 h at 85 V and revealed on a UV table after GelRed^®^ staining.

### 2.4. MALDI-TOF MS for Typing

For MALDI-TOF MS typing (T-MALDI), the spectra of the strains acquired by MALDI-TOF MS were analysed by ClinProTools software (version 3.0, Bruker) in order to classify each strain in a specific cluster based on a machine learning method, involving the Genetic Algorithm (GA) classifying algorithm model (CAM) previously developed and described [16].

The analysis was focused on the molecular mass range 2–20 kDa, with a 7.5 signal-to-noise ratio and a 0.75 noise threshold. All spectra were automatically re-calibrated, with “Shift Maximum Peak” set up at 1000 ppm to reduce the mass shifts that could arise during multiple acquisitions.

All the spectra obtained for all the strains analysed with T-MALDI were classified with the CAM previously created with the 5 predominant ribotypes circulating in the same University Hospital in the 2 previous years [16]. This CAM contained Ribotype 126, Ribotype 018, and 3 ribotypes arbitrarily (PR2, PR4, and PR5), named and not referable to an official ribotype. For each strain, 10 spectra were classified with the CAM; if more than 8 spectra were classified in the same cluster a classification was considered reliable, if 7–8 spectra were classified in the same cluster the classification was considered “low reliable”, if 1–6 spectra were classified in the same cluster the classification was considered unreliable.

In addition, these spectra were analysed by Principal Component Analysis (PCA), an unsupervised hierarchical type of clustering, in order to visualize the homogeneity and heterogeneity of the protein spectra. The PCA results are called scores and are derived and displayed in various plots. The score output represents the original data mapped into the new coordinate system, which is defined by the Principal Components (PCs). Within the score plot, outlier spectra from a group or from several groups can be discovered and visualized. The outliers are spectra that are extreme or do not fit the PCA model. Independently from the PC coordinates, the score plots contain the same spectra number as the original data set. Moreover, the percentage of the “explained variance” of the single given PC was also reported.

### 2.5. Reference Strains

The *C. difficile* 51377 strain (*tcd*A+/*tcd*B+/*cdt*+, Ribotype 127) [23] was used as positive control for *tcd*A, *tcd*B, and *cdt*A/*cdt*B detection. In addition, two strains, belonging to our collection and genetically characterized by Cardiff reference laboratory [24] as PCR-Ribotype 126 and 018, were used to compare PCR-Ribotyping and T-MALDI results.

### 2.6. Antimicrobial Susceptibility Testing (AST)

The AST for MIC evaluation of vancomycin, metronidazole and moxifloxacin of each *C. difficile* isolate was performed by gradient diffusion test (Liofilchem, Roseto degli Abruzzi, Italy) plating a 0.5 McFarland suspension in saline solution onto two Schaedler agar medium plates (Kima). AST was interpreted according to MIC breakpoint criteria of the 2022 CLSI [25].

## 3. Results

**Figure 1:**
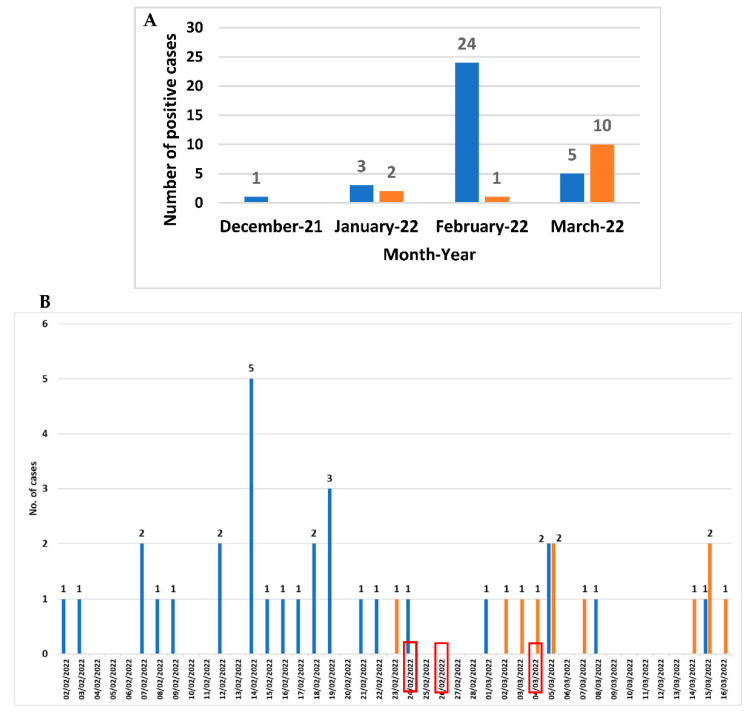
Month distribution from December 2021 to March 2022 (**A**) and daily distribution (**B**) from February to March 2022 of CDI cases in Long-term care Unit (blue) and in Internal Medicine ward (orange). The red boxes indicate the day when the three patients were transferred from the Long-term care Unit to the Internal Medicine ward.

From December 2021 to March 2022, among the 143 faecal samples belonging to 112 patients with suspicion of CDI, *C. difficile* DNA was revealed in 51 faecal samples belonging to 46 patients (30 females and 16 males; mean age 83 years ranging from 65 to 101 years), corresponding to a prevalence rate of 41.1% (46/112) (Figure 1A). The month distribution showed that a peak in February 2022 (25 cases) referred to a potential outbreak. Three out of the twenty-four *C. difficile*-positive patients attending the Long-term Care Unit were transferred to the Internal Medicine ward (the first one at the end of February). Since the beginning of March, the number of *C. difficile*-positive cases attending Internal Medicine ward began to increase (Figure 1B).

Based on the diagnostic algorithm used, on the overall 40 *C. difficile* DNA-positive patients (45 faecal samples) detected in February and March, GDH was revealed in 44 faecal samples belonging to 39 patients (97.5%; 39/40). Thirty-seven faecal samples from 32 patients were positive to toxins A/B (80%; 32/40). For 29 patients, a *C. difficile* strain was obtained by CC (72.5%; 29/40).

Among the 29 *C. difficile* strains isolated by CC, 28 grew sufficiently to be typed by T-MALDI and to be submitted to AST for vancomycin, metronidazole, and moxifloxacin.

**Figure 2:**
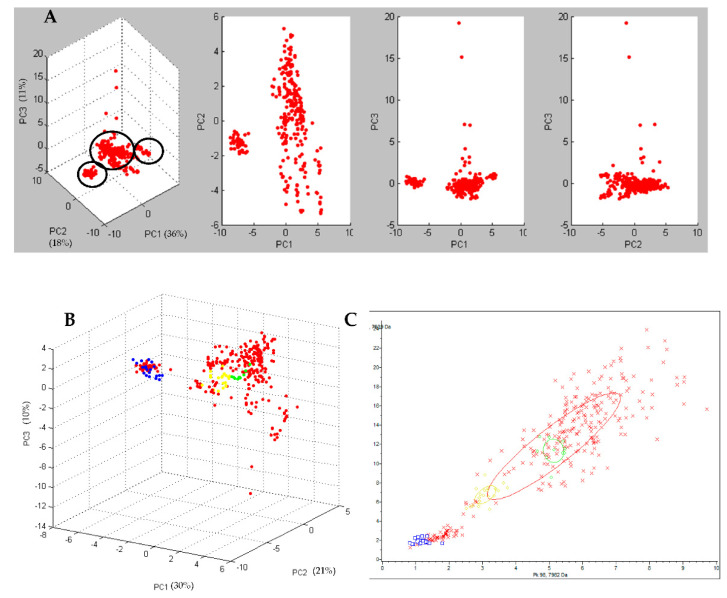
In (**A**) three-dimensional plot of all the 28 *C. difficile* strains, obtained by Principal Component Analysis (PCA) grouped in 3 different clusters (circled in black). Dots represents the single spectra. In (**B**) three-dimensional plot of all the 28 *C. difficile* strains (red), compared to Ribotype 126 (blue), Ribotype 018 (yellow), and Ribotype 127 (green). Dots of the same colour represent the single replicates of the same class. In (**C**) 2-D distribution of all the 28 *C. difficile* strains (red), compared to Ribotype 126 (blue), Ribotype 018 (yellow), and Ribotype 127 (green). The “x” of the same colour represents the single replicate of the same class.

By performing the PCA, the T-MALDI grouped the spectra of the 28 analyzed strains in three different clusters (Figure 2A). The cluster accounting for the greater number of strains (22/28; 78.6%) was defined as the principal cluster. The spectra of this cluster did not group with any of those obtained from the 3 reference Ribotypes (Ribotype 126, Ribotype 018, and Ribotype 127) used in this study. When these 22 strains were tested with the previously created CAM, 8 were classified as ribotype PR5, 6 as “low reliable” ribotype PR5, 2 as “low reliable” ribotype PR2, 1 as ribotype PR2, and the remaining 5 were not classified.

One of the two minor clusters, accounting for 4 strains (4/28; 14.3%), grouped with the spectra obtained for the reference Ribotype 126 (Figure 2B,C) by the PCA, and the strains were classified as Ribotype 126 by the previously created CAM.

The remaining cluster, accounting for 2 strains (2/28; 7.1%), did not group with any of the 3 reference Ribotypes by the PCA and only one of the 2 strains was classified (as ribotype PR5) with the previously created CAM.

DNA was extracted from all the 29 *C. difficile* isolates to perform PCR-Ribotyping and characterization for the presence of *tcd*A, *tcd*B, and *cdt*A/*cdt*B genes.

**Figure 3:**
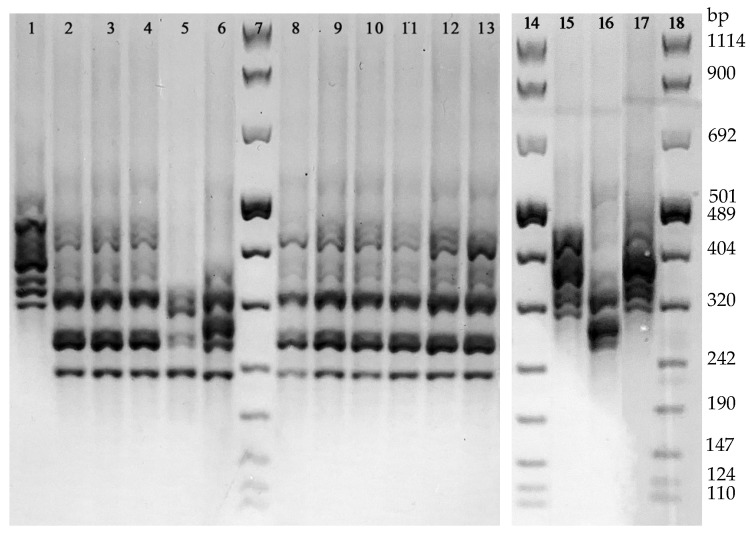
Different DNA amplification patterns obtained by PCR-Ribotyping. Line 1, Ribotype 126; Lines 2–4 and 8–13, ribotype PRA; Line 5, ribotype PRB; Line 6, rRibotype PRC; Line 15, Ribotype 127, Line 16, Ribotype 018; Line 17, Ribotype 126; Lines 7, 14, and 18, 100 bp DNA Molecular Weight Marker VIII.

With regard to PCR-Ribotyping, the analysis of the PCR amplification patterns revealed 4 different ribotypes. The 13.8% (4/29) of the isolates revealed a DNA amplification profile referring to the Ribotype 126 reference strain, while the remaining 25 strains were grouped in three arbitrarily named ribotypes: PRA (accounting for the 75.9%, 22/29, of the isolates), PRB (2 isolates), and PRC (1 isolate) (Figure 3). None of these latter 3 ribotypes showed a DNA amplification pattern referring to the reference Ribotypes used in this study (i.e., 018 and 127). The distribution of all the 29 isolates grouped in the 4 ribotypes according to their toxigenic and antibiotic susceptibility profiles is shown in Table 2.

The classification of the 4 strains as Ribotype 126 by T-MALDI was in agreement with that obtained by PCR-Ribotyping. Since any of the remaining 3 ribotypes found in this study (PRA, PRB, and PRC) did not show a DNA amplification profile corresponding to that of the ribotypes included in the CAM, the classification of the 18 strains by T-MALDI diverged from that observed by PCR-Ribotyping.

**Figure 4:**
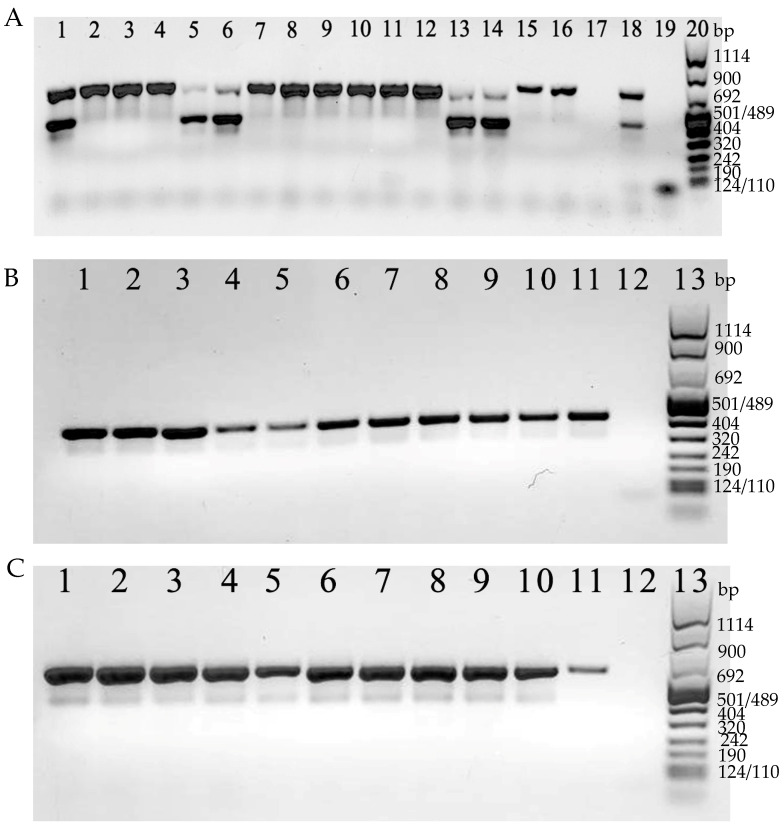
(**A**). Examples of detection of *tcd*A (624 bp) and *tcd*B (421 bp). Lines 1–16: *C. difficile* strains tested; line 17: *C. difficile tcd*A and *tcd*B negative control; line 18: *C. difficile tcd*A and *tcd*B positive control; line 19: negative PCR control (no DNA); line 20: 100 bp DNA Molecular Weight Marker VIII. In (**B**), examples of detection of *cdt*A (375 bp). Lines 1–10 *C. difficile* strains tested; line 11: *C. difficile cdt*A positive control; line 12: *C. difficile cdt*A negative control; line 13: 100 bp DNA Molecular Weight Marker VIII. In (**C**), examples of detection of *cdt*B (510 bp). Lines 1–10 *C. difficile* strains tested; line 11: *C. difficile cdt*B positive control; line 12: *C. difficile cdt*B negative control; line 13: 100 bp DNA Molecular Weight Marker VIII.

Both toxin A and B genes were revealed together (*tcd*A+/*tcd*B+) in 7 isolates (24.1%; 7/29), whereas in the remaining 22 isolates (75.9%; 22/29), only the toxin A gene was detected (*tcd*A+/*tcd*B−) (Figure 4A).

On the other hand, CDT genes were revealed in 26 isolates (89.7%; 26/29). For the remaining 3 isolates (*tcd*A+/*tcd*B+), CDT genes were not detected (Figure 4B,C).

Overall, 22 strains (75.9%; 22/29) *tcd*A+/*tcd*B−/*cdt*+, 4 strains (13.8%; 4/29) *tcd*A+/*tcd*B+/*cdt*+, and 3 strains (10.3%; 3/29) *tcd*A+/*tcd*B+/*cdt*−, were found (Table 2).

All the 28 *C. difficile* isolates tested for antimicrobial susceptibility were found to be susceptible to vancomycin (MIC range from 0.032 mg/mL to 0.5 mg/mL) and metronidazole (MIC range from 0.125 mg/mL to 0.25 mg/mL). On the contrary, all strains were not susceptible to moxifloxacin (MIC > 32 mg/mL), except three strains (MIC range from 0.5 mg/mL to 1 mg/mL) and binary toxin non-producer (*tcd*A+/*tcd*B+/*cdt*−) (Table 2).

## 4. Discussion

Antibiotic therapy and hospitalization are the two main risk factors for CDI, especially for the elderly population who prove to be more susceptible [26]. The epidemiological scenario of CDI in hospital settings underwent a significant change during the last two years due to the emergence of the novel Coronavirus disease (COVID-19). According to the epidemiological data available from January 2019 to September 2021, a decreasing trend of hospital-acquired CDI has been registered during the COVID-19 pandemic in Long-term Care Facilities [27,28,29,30]. Although the misuse and overuse of broad-spectrum antibiotics to prevent bacterial co-infections and super-infections exposed the COVID-19 patients to an increased risk to develop CDI, the control measures and the cleaning regimens (barrier precautions, increased focus on hygiene, environmental cleaning, patient isolation, and the increased use of personal protective equipment) could have played an important role in preventing *C. difficile* transmission [31].

This study reports an outbreak of *C. difficile* occurred in two units of the University Hospital of Parma (Italy) for two months (from February to March 2022) involving 40 toxigenic *C. difficile* DNA-positive patients: 29 hospitalized in a Long-term Care Unit and 11 attending an Internal Medicine ward, where three *C. difficile*-positive patients initially admitted to LCI were transferred.

Among the 40 *C. difficile* DNA-positive patients, *C. difficile* was isolated in 29 cases (22 from Long-term Care Unit and 7 from the Internal Medicine ward), which were submitted to genotypic characterization. Out of the 29 strains, 22 belonged to the same cluster (PRA) and were linked to the outbreak: 17 have been isolated from the 22 patients admitted to the Long-term Care Unit and 5 from the 7 admitted to the Internal Medicine ward. All these epidemic strains were susceptible to vancomycin (MIC ≤ 0.5 mg/mL) and metronidazole (MIC ≤ 1 mg/mL), but not susceptible to moxifloxacin (MIC > 32 mg/mL) and produced only one (Toxin A) of the major large toxins and, additionally, the binary toxin (*tcd*A+/*tcd*B−/*cdt*+). The A+B- phenotype has rarely been reported in literature [32] and mostly isolated in animals, such as cattle [12].

To our knowledge, this is the first reported outbreak related to a *tcd*A+/*tcd*B−/*cdt*+ toxigenic profile. Strains carrying such a feature are not commonly reported, however a *tcd*A+/*tcd*B−/*cdt*+ toxigenic profile has already been described in literature and characterized as Ribotype 033 [12,32]. Despite its positivity for the *tcd*A gene using the PCR assay indicated by ECDC [12], the Ribotype 033 does not produce an active Toxin A due to an extensive deletion outside the *tcd*A region amplified by the PCR above mentioned, thus resulting negative when analysed by immunoenzimatic assays for this toxin [12]. On the contrary, in our study, immunochromatographic assays resulted as positive in the majority of the cases, leading us to exclude that the involved strains belong to the Ribotype 033.

Therefore, it is reasonably assumable that the *tcd*B gene negative outcomes obtained by PCR in this study could be due to changes into the *tcd*B region targeted for the amplification. Accordingly, A+B− strains could carry variant forms of *tcd*B and actually belong to one of the known A+B+ variant toxinotypes [32].

The remaining 7 strains were grouped in 3 different Ribotypes 126, PRB, and PRC, among which the most representative one (4 strains) shared the same Ribotyping and toxigenic profiles of the reference Ribotype 126. On the other hand, according to the comparative analysis of the DNA amplification patterns, the PRB and PRC types were different from any of the reference Ribotyped strains used in the study, including Ribotype 018, which has been the most prevalent nosocomial Ribotypes isolated in Italy since 2006 [12].

With regard to the T-MALDI for typing strains, a significant capability in discovering a potential outbreak has been demonstrated by the unsupervised PCA which grouped the spectra of the 28 *C. difficile* strains in three different clusters. The most representative cluster accounted the greater number of acquired spectra and turned out to be composed of the *C. difficile* strains typed as PRA by PCR-Ribotyping, namely the epidemic cluster.

Moreover, the epidemic cluster did not match with any of the clusters of the three reference strains; however, a correlation between the Ribotype 126 cluster and one of the two non-epidemic cluster was found. These results were in agreement with those of PCR-Ribotyping that excluded the occurrence of the 018 and 127 reference strains.

T-MALDI correctly classified the spectra correlated to the Ribotype 126 cluster that was included in the CAM. On the other hand, since the CAM previously created [16] did not include the Ribotype PRA, the T-MALDI, as expected, failed in classifying the strains included in such epidemic cluster. This faulty result highlights the difficulties of the T-MALDI in typing strains not included in the CAM, given the potential genetic variability of *C. difficile* strains within the same lineage and the spread of new circulating ribotypes, as emerged in this study [16].

The ongoing increase of the number of ribotypes belonging to a specific lineage [12] could give reason to the difficulties of the T-MALDI approach in classifying new ribotypes genetically related. It is worth noting that T-MALDI relies on an algorithm approach, allowing the differentiation of protein profiles on the basis of just one discriminating peak [16], whereas a genetic homology of ≥80% between DNA amplification pattern of at least two different strains is enough to ribotype them within the same lineage [12].

In the light of the results obtained in this study, T-MALDI is a valid and rapid approach in discovering and tracking outbreaks in healthcare settings. However, the development of a CAM requires a high number of different ribotypes to specifically and correctly classify strains of clinical and epidemiological relevance. As a matter of fact, the machine learning combined with MALDI-TOF MS would prove to be a powerful tool at the service of epidemiological surveillance centres involved in epidemiological tracking of CDI worldwide which, as such, would receive a great number of different ribotypes and isolates/ribotype. A correct performance of the CAM requires its continuous updating on the basis of epidemiological data in order to include new unknown circulating ribotypes.

## Figures and Tables

**Table 1:** Demographic information of the 112 patients whose samples were analysed.

Ward	Age	Sex	No.
IM	60–70	F	2
	70–80	F	7
		M	4
	80–90	F	4
		M	6
	90–100	F	4
		M	3
	>100	F	1
LT	30–40	F	1
	40–50	M	1
	50–60	F	1
		M	2
	60–70	F	6
		M	5
	70–80	F	13
		M	8
	80–90	F	23
		M	9
	90–100	F	10
		M	2
Total			112

IM: Internal Medicine ward; LT: Long-term Care Unit; F: female; M: male.

**Table 2:** Characterization of the 29 *C. difficile* isolates.

Strain No.	Ward (No.)	T-MALDI (No.)	Ribotype (No.)	Gene Detection			SIR (MIC Range)		
				*tcd*A (No.)	*tcd*B (No.)	*cdt*A/*cdt*B (No.)	Vancomycin	Metronidazole	Moxifloxacin
4	LT (4)	RT 126 (4)	RT 126 (4)	Positive (4)	Positive (4)	Positive (4)	S (0.25–0.5)	S (0.125–0.25)	R (>32)
17	LT (17)	NP (1)	PR A (22)	Positive (22)	Negative (22)	Positive (22)	NP	NP	NP
		PR5 (7)					S (0.032–0.5)	S (0.125–0.25)	R (>32)
		PR5 LRC (4)							
		NRC (3)							
		PR2 (1)							
		PR2 LRC (1)							
5	IM (5)	PR5 LRC (2)							
		NRC (2)							
		PR5 (1)							
2	LT (1)	PR5 (1)	PR B (2)	Positive (2)	Positive (2)	Negative (2)	S (0.5)	S (1)	S (0.5)
	IM (1)	NRC (1)							
1	IM (1)	PR2 LRC (1)	PR C (1)	Positive (1)	Positive (1)	Negative (1)	S (0.5)	S (1)	S (0.5)

SIR: MIC interpretive categories, S: susceptible, R: resistant, LT: Long-term Care Unit; IM: Internal Medicine ward; LRC: low reliable classification; NRC: Not reliable classification; NP: Not performed.

## Data Availability

The data presented in this study are available in the manuscript.
